# The Power of Color Flow Doppler Ultrasonography Versus Blind Technique in Localization of Epidural Catheter: A Randomized Prospective Study

**DOI:** 10.5812/aapm-147828

**Published:** 2024-06-30

**Authors:** Atef Mohamed Mahmoud, Safaa Gaber Ragab, Taha Mohamed Agamy, Abeer Shaban Goda

**Affiliations:** 1 Department of Anesthesia, Faculty of Medicine, University of Fayoum, Faiyum, Egypt; 2 Faculty of Medicine, University of Fayoum, Faiyum, Egypt

**Keywords:** Ultrasonography, Doppler, Catheter, Analgesia

## Abstract

**Background:**

The success of epidural analgesia hinges on the precise insertion of the needle within the epidural space; failure rates have been reported to reach 32%.

**Objectives:**

We report a new method using color Doppler to help verify the accurate location of the epidural needle tip.

**Methods:**

This is a randomized prospective study. Sixty patients undergoing hysterectomy were enrolled, with 30 patients in each group. Color flow Doppler (CFD) ultrasonography was employed to guide epidural catheter placement. The ultrasound-guided epidural technique was used for patients where challenges in identifying traditional landmarks for epidural space localization were anticipated. The procedure commenced with a spinal epidural technique. After sterile preparation and draping of the area, a curvilinear ultrasound transducer, encased in a sterile sheath, was used to locate the interspinous space. The primary outcome measure focused on flow visualization at different insertion levels. Secondary outcome measures included the duration of catheter implantation, intervertebral level of insertion, and dermatome sensory levels. The study also assessed the quality of epidural analgesia and patients' assessment of analgesic quality using a Verbal Numerical Rating Scale.

**Results:**

The study reported a successful and predominantly safe outcome, with high success rates in flow visualization and effective anesthesia coverage. Flow visualization at the insertion and surrounding levels demonstrated a 100% success rate at all observed points. The Visual Numeric Rating Scale (VNRS) results indicated a median pain score of 2 with an interquartile range (IQR) of 2 - 3, showcasing a generally low level of post-procedural pain among the subjects, reflecting good quality post-operative analgesia.

Regarding dermatome sensory levels after 2 hours, the distribution across various levels, including T4, T6, T7, T8, T10, and T12, exhibited a favorable outcome. The highest proportion was observed at T10 (68.3%), suggesting effective anesthesia coverage in the targeted areas. The study demonstrated comparable efficiency between the CFD-guided and blind techniques in terms of procedural aspects. However, notable distinctions were observed in patients' reported pain levels, with the CFD group experiencing lower pain compared to the blind technique group. Additionally, the study highlighted the association between CFD and improved procedural accuracy and safety.

**Conclusions:**

This study advocates for the integration of CFD into routine clinical practice to enhance procedural outcomes and patient safety during hysterectomy surgeries.

## 1. Background

Cesarean sections and hysterectomies are the primary surgical operations in gynecology (1). Benign uterine diseases, including endometriosis and uterine fibroids, can lead to irregular bleeding, causing anemia and reducing work capability and quality of life, similar to malignant uterine diseases (2). Color flow Doppler (CFD) imaging has significantly improved ultrasonography (US) by encoding the mean Doppler frequency shift to display local flow. Though the selection of mean frequency shift as the parameter is somewhat arbitrary, this approach enhances precision. Doppler ultrasound is a noninvasive test that measures blood flow through blood vessels by bouncing high-frequency sound waves off red blood cells circulating in the bloodstream. While a regular ultrasound uses sound waves to produce images, it cannot show blood flow (3). US improves accuracy in identifying optimal epidural puncture sites, reducing attempts compared to the loss of resistance test (4). Its advantages are notable in challenging cases like scoliosis, prior spinal surgery, or major surgeries such as hysterectomies (5). 

Color flow Doppler is primarily used for imaging blood flow in different organs, arteries, and veins. It is a common feature in ultrasound machines, proving valuable in various clinical conditions and operations. While similar to obtaining anatomical information, obtaining velocity information is technically more demanding (6). Color flow Doppler has recently been used to decide the placement of epidural catheters. Ultrasonography in M-mode has been reported to be useful for locating epidural catheters (7). It is anticipated that precise identification of the epidural area will lower the incidence of complications such as unintentional dural puncture, which occurs in 0.19% to 3.6% of cases following epidural implantation in labor (8, 9). This demonstrates the significance of position identification to avoid complications. The positioning of epidural catheters is frequently facilitated by the use of ultrasound. However, data on its ability to validate its location in the epidural space are scarce, and it is still unknown how CFD applies to epidural anesthesia for hysterectomy.

## 2. Objectives

The study's main objective is to use CFD ultrasonography versus the blind technique to precisely locate and validate the epidural catheter's placement during hysterectomy surgeries. In the context of epidural anesthesia for hysterectomy (10), this aims to improve the accuracy of catheter location, contributing to better procedural outcomes and patient safety.

## 3. Methods

### 3.1. Aim, Design, and Setting

This randomized prospective study aimed to employ color flow Doppler ultrasonography versus the blind technique for precise localization and validation of epidural catheter placement during hysterectomy surgeries. The research was conducted at Fayoum University Hospital in Egypt, following approvals from the local Institutional Ethics Committee and the institutional review board of the Faculty of Medicine at Fayoum University. The study setting, within the confines of Fayoum University Hospital, provided an ideal backdrop for a comprehensive investigation.

### 3.2. Study Participants

Sixty patients were inclusively enrolled in the study. Each participant's data encompassed demographics and dermatome level. The spinal-epidural method was the selected procedure for data acquisition.

### 3.3. Ethical Considerations

The research, conducted under number M649, received approval from the local Institutional Review Board and Institutional Ethics Committee of the Faculty of Medicine at Fayoum University. Rigorous adherence to ethical standards was ensured, with each eligible patient signing a comprehensive informed consent form before their inclusion in the study.

### 3.4. Clinical Trials Registration

The study is registered with ClinicalTrials.gov under the identifier NCT06019039, providing transparency and accessibility to the trial details, and was first posted on 31/08/2023.

### 3.5. Randomization

Randomization was performed using a computer-generated randomization sequence by an independent researcher not involved in patient recruitment or data collection. Allocation concealment was ensured through the use of sequentially numbered, opaque, sealed envelopes (SNOSE) containing the treatment allocation. Patients were randomly assigned in a 1: 1 ratio to either the color flow Doppler (CFD) group or the blind technique group. The randomization process was conducted prior to the start of the study and was overseen by the principal investigator to maintain integrity and transparency. The data collector and assessor remained blinded to the treatment allocation throughout the study duration.

#### 3.5.1. Inclusion Criteria

- Women undergoing hysterectomy under epidural analgesia.

- Women with an epidural catheter still in place.

- American Society of Anesthesiologists (ASA) physical status I or II.

- Age range: 30 to 70 years old.

#### 3.5.2. Exclusion Criteria

- Patient refusal.

- History of allergy to one of the study drugs.

- Any contraindication to regional anesthesia, such as local infection or bleeding disorders.

- Patients with a history of spine disorders, such as scoliosis or previous spine surgery.

- Obesity, defined as a Body Mass Index (BMI) of ≥ 35 kg/m^2^.

### 3.6. Primary Outcome Measures

Flow Visualization in Relation to Interspace Insertion Level Using Color Flow Doppler Ultrasonography:

- No Flow Visualization: At Insertion Level: [Yes/No], Above Insertion Level: [Yes/No], Below Insertion Level: [Yes/No]

### 3.7. Secondary Outcome Measures

- Duration of Epidural Catheter Insertion: The duration of catheter insertion will be assessed, as it demonstrates good localization and proper placement of the catheter.

- Questionnaire: Intervertebral Level of Epidural Insertion: Anesthesiologist documentation will determine whether the epidural was inserted at the L2 - L3 or L3 - L4 intervertebral level.

- Questionnaire: Maximum Upper Sensory Block to Ice: The nurse will record the highest sensory block level to ice in the computerized charting system (2 hours post-operative assessment).

- Patient's Assessment of Analgesic Quality: Using a Verbal Numerical Rating Scale (VNRS) ranging from 0 to 10, patients will assess the quality of analgesia once, 2 hours post-operatively, to determine the quality, not the duration, as the main focus of this study is the proper localization of epidural catheter placement.

- Patient Characteristics: Age, weight, height, and BMI.

- Complications: Including postdural puncture and false loss of resistance.

### 3.8. Procedure

After entering the operating room, intravenous access and monitoring, including ECG, pulse oximeter, and ABP, were established for the patients. Resuscitation equipment was available for any problems that might occur during the procedure. After the patient was seated and aseptic precautions were taken, 2 mg of midazolam and 20 μg of fentanyl were administered. The ultrasound-guided epidural technique was employed for patients where challenges in identifying traditional landmarks for epidural space localization were anticipated.

Following meticulous sterile preparation of the area, a sterile sheath housing a curvilinear ultrasound transducer was carefully inserted. The procedure commenced with a spinal epidural technique. After sterile preparation and draping of the area, a curvilinear ultrasound transducer, encased in a sterile sheath, was used to locate the interspinous space. The epidural needle was then guided under ultrasound direction, utilizing an out-of-plane technique with a two-hand method to manipulate both the needle and the ultrasound probe. Advancement of an 18-G Tuohy needle proceeded until the loss of resistance to normal saline was achieved. Verification of the epidural space using CFD followed the injection of up to 8 mL of normal saline via the epidural needle. Subsequently, a 21-G epidural catheter was inserted into the epidural area. 

To verify successful epidural placement, a 22-G, 50 mm needle was introduced until cerebrospinal fluid (CSF) was observed. A local anesthetic was then administered as a bolus dose intrathecally. All CFD images were acquired using a Phillips CX50 ultrasound machine equipped with a 5.0 MHz low-frequency curvilinear US probe (LFC_50, 15_6MHz) from Phillips. A two-dimensional transverse interspinous view was employed to visualize the posterior complex (ligamentum flavum, epidural space, and dura).

Upon achieving the loss of resistance to normal saline, the CFD function was activated to detect flow via the epidural needle tip. The ultrasound probe was placed in the transverse axis just below the epidural needle but within the interspinous space. Optimal imaging was ensured through adjustments to scanning depth (6 - 12 cm), frequency range, gain, and time-gate compensation. The color flow Doppler was utilized to enhance qualitative flow depiction. The direction and inclination of the ultrasound probe were carefully adjusted for the best visualization, with a preference for an upward tilt parallel to the lumbar spinous process orientation. During CFD recording, 5 mL of sterile saline was administered as a flush via the needle. The same procedure was repeated with a slow injection of 5 mL of saline, with the administration flow left non-standardized. The coloration scale was calibrated to augment color flux while avoiding excessive aliasing, preferably within the range of 12 - 20 without the need for baseline adjustment.

After epidural catheter placement, parasagittal and transverse views with CFD were acquired to identify the catheter's location within the epidural area. Two levels above and below the introduction site were examined using the previously described CFD settings. Saline flow via the catheter was visualized as a blue and red mosaic, as the signal aliased from one color to the next, aiding in spotting the pathway of the catheter and its end position. If color waves were not visualized on one side of the vertebrae, the PO view was likewise obtained on the other side. If no waves were detected at the level of catheter introduction on each side, the imaging intervention was repeated at one or, at most, two spinal levels above and below the site of catheter introduction on each side of the median plane of the body (midline).

### 3.9. Sample Size

The sample size was calculated using G-Power© software version 3.1.7 (Institute of Experimental Psychology, Heinrich Heine University, Düsseldorf, Germany). Based on the guidance of a previous study (11), the minimum sample size was determined to be 60 patients. The effect size was 0.47, depending on previous research results. The calculation assumed a two-sided (two-tailed) type I error of 0.05 and a power of 80%.

### 3.10. Statistical Analysis

Statistical analysis was conducted using SPSS version 27 (IBM Co., Armonk, NY, USA). Quantitative parametric data were presented as mean and standard deviation (SD) and analyzed using the independent samples Student *t*-test. Quantitative non-parametric data were presented as median and interquartile range (IQR) and analyzed using the Mann–Whitney test. Categorical data were presented as frequency and percentage and analyzed using the chi-square test or Fisher's exact test when appropriate. A two-tailed P-value < 0.05 was considered statistically significant.

## 4. Results

**Figure 1.**
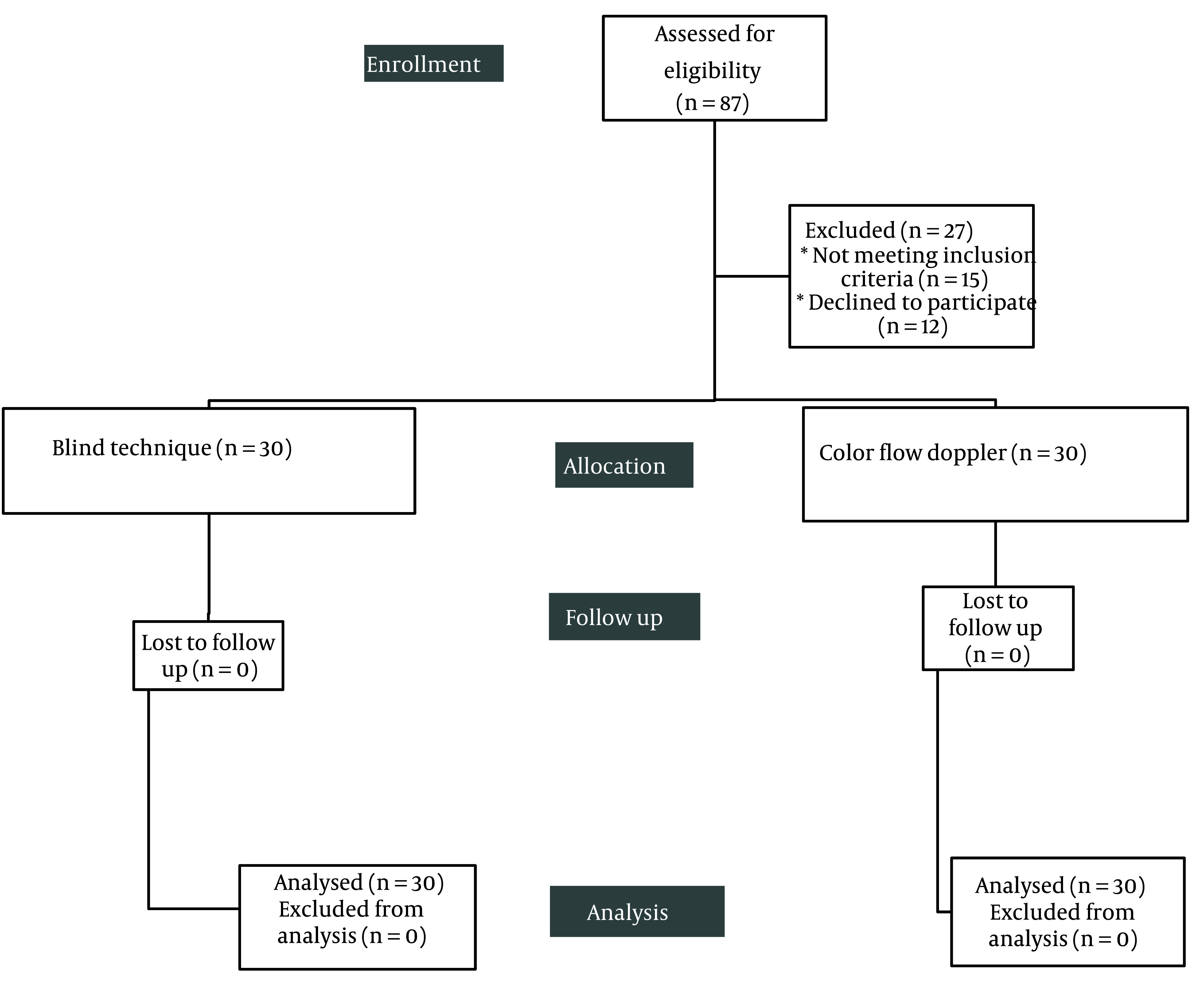
CONSORT flowchart

Sixty female patients who underwent hysterectomy were allocated into two groups based on the technique used for the localization of the epidural catheter: 30 patients with color flow Doppler technique and 30 patients with the blind technique. All patients were followed up and analyzed statistically (Figure 1). 

Sixty patients underwent hysterectomy and localization of epidural catheter was done by color flow Doppler technique in 30 (50%) patients and by blind technique in 30 (50%) patients. The height ranged from 155 to 180 cm, with a median value of 170 cm. BMI had a mean value (± SD) of 28.77 (± 2.29) kg/m^2^, ranging from 24 to 37 kg/m^2^ (Table 1). 

**Table 1.** Characteristics of the Studied Patients (N = 60) ^a^

Group	Values
**Color flow Doppler**	30 (50)
**Blind technique**	30 (50)
**Age (y)**	53.92 ± 8.1
**Weight (kg)**	83.92 ± 7.88
**Height (cm) Median (IQR)**	170 (168.25 - 175)
**BMI**	28.77 ± 2.29

Abbreviation: BMI, Body Mass Index.

^a^ Values are expressed as Mean ± SD or No. (%).

The results of the epidural insertion procedure at different levels indicate a successful and predominantly safe outcome. The majority of insertions occurred at the L3 - L4 and L4 - L5 levels, with 46.7% and 53.3%, respectively. The flow visualization at insertion and surrounding levels demonstrated a high success rate, achieving 100% at all observed points. Regarding dermatome sensory levels after 2 hours, the distribution across various levels, including T4, T6, T7, T8, T10, and T12, exhibited a favorable outcome. Notably, the highest proportion was observed at T10 (68.3%), suggesting effective anesthesia coverage in the targeted areas.

The Visual Numeric Rating Scale (VNRS) results indicated a median pain score of 2 with an interquartile range (IQR) of 2 - 3, showcasing a generally low level of post-operative pain 2 hours after the procedure among the subjects. Complication rates were notably low, with only one case of dural puncture (1.7%) and a small incidence of false loss of resistance (11.7%). These findings reflect a high degree of procedural accuracy and safety. The duration of epidural insertion varied, with 20.0%, 26.7%, and 53.3% of insertions lasting 24 hours, 36 hours, and 48 hours, respectively (Table 2). 

**Table 2.** Operative Details of the Studied Patients (n = 60)

Level of Insertion	No. (%)
**L3 - L4**	28 (46.7)
**L4 - L5**	32 (53.3)
**Visualization of flow**	30 (50)
**Visualization of flow (out of 30)**	
At insertion level	30 (100.0)
Above insertion level	30 (100.0)
Below insertion level	3 (10.0)
**Dermatome sensory level after 2 hrs. post-operative**	
T4	2 (3.3)
T6	3 (5.0)
T7	1 (1.7)
T8	11 (18.3)
T10	41 (68.3)
T12	1 (1.7)
T12_L1	1 (1.7)
**VNRS after 2hr post-operative**	
**Median (IQR)**	2 (2 - 3)
**Dural puncture**	1 (1.7)
**False loss of resistance**	7 (11.7)
**Duration of epidural insertion**	
24 h	12 (20.0)
36 h	16 (26.7)
48 h	32 (53.3)

No statistically significant differences were observed in age, weight, or BMI between the two techniques. The mean age for the color flow Doppler group was 52.66 years with a standard deviation of 8.47, while the Blind Technique group had a mean age of 55.10 years with a standard deviation of 7.69 (P = 0.247). Likewise, there were no significant differences in weight (P = 0.708) and BMI (P = 0.758) between the color flow Doppler and Blind Technique groups. The mean weight for the color flow Doppler group was 83.52 kg (SD = 7.83), and for the Blind Technique group, it was 84.29 kg (SD = 8.05). The mean BMI for the color flow Doppler group was 28.86 (SD = 1.79), and for the Blind Technique group, it was 28.68 (SD = 2.70).

Interestingly, a significant difference was observed in height between the two techniques (P = 0.042). The median height for the color flow Doppler group was 170 cm with an interquartile range (IQR) of 167-172.5 cm, while the Blind Technique group had a median height of 173 cm with an IQR of 169-175 cm. This suggests that individuals undergoing the Blind Technique tended to be taller compared to those undergoing color flow Doppler. In conclusion, while age, weight, and BMI did not differ significantly between the color flow Doppler and Blind Technique groups, a notable difference in height was observed (Table 3). 

**Table 3.** Association of Technique used in Localization of Epidural Catheter with Characteristics of the Studied Patients

Group	N	Values	P-Value	95% C.I. of the Difference
**Age (y)**			0.247	-6.62 to 1.73
Color flow Doppler	30	52.66 ± 8.47		
Blind technique	30	55.10 ± 7.69		
**Weight(kg)**			0.708	-4.88 to 3.33
Color flow Doppler	30	83.52 ± 7.83		
Blind technique	30	84.29 ± 8.05		
**BMI**			0.758	-1.01 to 1.38
Color flow Doppler	30	28.86 ± 1.79		
Blind technique	30	28.68 ± 2.70		
**Height**			0.042 ^a^	-
Color flow Doppler	30	170 (167-172.5)		
Blind technique	30	173 (169-175)		

Abbreviation: BMI, Body Mass Index.

^a^ Significant when P-Value < 0.05.

^b^ Values are expressed as Mean ± SD or Median (IQR).

**Figure 2.**
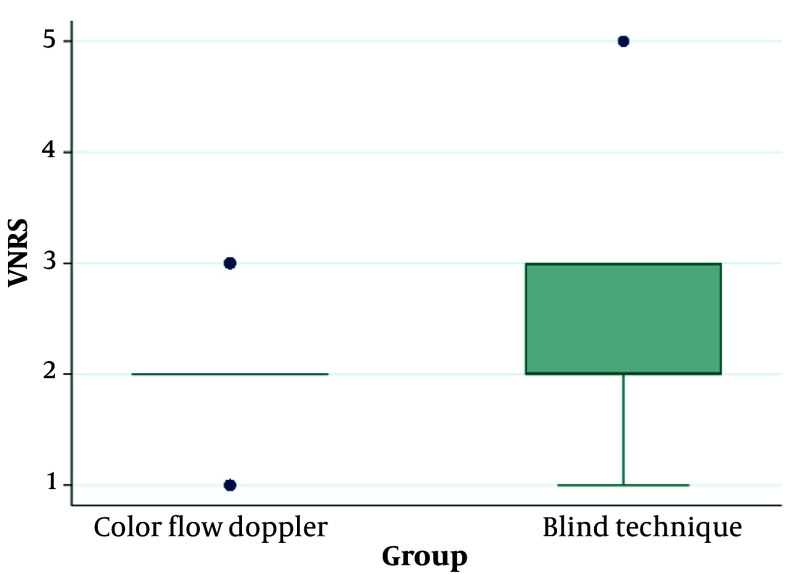
VNRS of the studied groups

Regarding the Verbal Numerical Rating Scale (VNRS) to assess pain levels, significant differences were found between the color flow Doppler and Blind Technique groups. The median pain rating for individuals undergoing color flow Doppler was 2, with an interquartile range (IQR) of 2 to 3, corresponding to a mean rank of 25.6. In contrast, the Blind Technique group had a median pain rating of 2, with a narrower IQR of 2 to 2, resulting in a higher mean rank of 35.06. The observed P-value of 0.015 indicates statistical significance (P < 0.05), suggesting that individuals undergoing color flow Doppler reported lower pain levels compared to those undergoing the Blind Technique. This finding highlights the potential impact of procedural technique on patient-reported pain outcomes, as assessed through the VNRS. The use of color flow Doppler appears to be associated with lower perceived pain levels compared to the Blind Technique (Table 4 and Figure 2). 

**Table 4.** Association of Technique used in Localization of Epidural Catheter with VNRS of the Studied Patients

Group	N	Median	IQR	Mean Rank	P-Value
**VNRS after 2 h post-operative**					
Color flow Doppler	30	2	2 (3)	25.6	0.015*
Blind technique	30	2	2 (2)	35.06	

Abbreviation: VNRS, verbal numerical rating scale.

^a^ Significant when P-value < 0.05.

In the comparison of procedural aspects between the color flow Doppler and Blind Technique groups, several noteworthy findings emerged. The level of insertion, specifically at L3 - L4 and L4 - L5, did not demonstrate a significant difference between the two techniques (P = 0.782). Approximately 44.8% of individuals in the color flow Doppler group and 48.4% in the Blind Technique group had their epidural inserted at the L3 - L4 level, while 55.2% in the color flow Doppler group and 51.6% in the Blind Technique group had insertion at the L4 - L5 level.

The dermatome sensory level after 2 hours post-procedure showed significant differences between the two groups (P < 0.001). Individuals in the color flow Doppler group exhibited a lower incidence of sensory loss at various dermatome levels, such as T4, T6, T7, T8, T10, and T12, compared to the Blind Technique group. Notably, the color flow Doppler group had no reported sensory loss at the T12 and T12_L1 levels, whereas the Blind Technique group had incidences of sensory loss at these levels.

Regarding procedural complications, there was no significant difference in the occurrence of false loss of resistance between the color flow Doppler and Blind Technique groups (P = 0.426). The majority of participants in both groups experienced no false loss of resistance, with 93.1% in the color flow Doppler group and 83.9% in the Blind Technique group reporting no occurrence. Similarly, no significant difference was observed in the incidence of dural puncture between the color flow Doppler and Blind Technique groups (P > 0.999). The overwhelming majority of participants in both groups did not experience dural puncture, with 100% in the color flow Doppler group and 96.8% in the Blind Technique group reporting no occurrence.

Finally, the duration of epidural insertion at different time intervals (24, 36, and 48 hours) did not show a significant difference between the color flow Doppler and Blind Technique groups (P = 0.440). The distribution of duration was relatively comparable between the two techniques at each specified time interval.

In summary, while the level of insertion and certain procedural aspects did not differ significantly between the color flow Doppler and Blind Technique groups, notable distinctions were observed in the dermatome sensory level after 2 hours post-procedure (Table 5). 

**Table 5.** Association of Technique Used in Localization of Epidural Catheter with Characteristics of the Operation of the Studied Patients

Variables	Group		P-Value
**Color Flow Doppler**	**Blind Technique**
**Level of insertion**			0.782
L3 - L4	14 (46.7)	14 (46.7)	
L4 - L5	16 (53.3)	16 (53.3)	
**Dermatome sensory level after 2 hrs.**			< 0.001
T4	2 (6.7)	0 (0)	
T6	2 (6.7)	1 (3.3)	
T7	1 (3.3)	0 (0)	
T8	10 (33.3)	1 (3.3)	
T10	15 (50)	26 (87.7)	
T12	0 (0)	1 (3.3)	
T12_L1	0 (0)	1 (3.3)	
**False loss of resistance**			0.426
No	28 (93.3)	25 (83.3)	
Yes	2 (6.7)	5 (16.7)	
**Dural puncture**			> 0.999
No	30 (100)	29 (96.7)	
Yes	0 (0)	1 (3.3)	
**Duration of epidural insertion**			0.440
24	8 (26.7)	4 (13.3)	
36	9 (30)	7 (23.3)	
48	13 (43.3)	19 (63.3)	

## 5. Discussion

The results of this prospective study exploring the use of color flow Doppler (CFD) ultrasonography for precise localization and validation of epidural catheter placement during hysterectomy surgeries reveal several significant findings. Notably, the age, weight, and Body Mass Index (BMI) of patients did not exhibit statistically significant differences between the CFD and Blind Technique groups, indicating a balanced distribution of demographic characteristics. The observed difference in height between the two groups, while statistically significant, may have limited clinical relevance given the relatively small magnitude of the disparity.

The successful and predominantly safe outcome of the epidural insertion procedure is underscored by the high success rate of flow visualization at insertion and surrounding levels, as well as the favorable distribution of dermatome sensory levels 2 hours post-procedure. The use of CFD appears to contribute to effective anesthesia coverage, as evidenced by the majority of insertions occurring at the L3 - L4 and L4 - L5 levels and the lower reported pain levels in the CFD group compared to the Blind Technique group. The utilization of CFD in the ultrasound-guided epidural technique demonstrated its efficacy in enhancing procedural accuracy and safety. The low rates of complications, including dural puncture and false loss of resistance, further support the feasibility and safety of this approach. The duration of epidural insertion, examined at different time intervals, displayed no significant differences between the CFD and Blind Technique groups, suggesting comparable efficiency in both techniques.

An increasing number of lumbar spine anatomical landmarks are being identified using two-dimensional ultrasound, which is also being used to place the epidural needle in real time (12, 13). While the ultrasound-identified distance between the skin and the posterior complex helps with the assessment of needle depth (14), basic ultrasonography may be challenging in people with abnormal spine anatomy. Rather than focusing on the expected depth of the epidural space, we employed CFD to directly identify the epidural space in our study (15). The release of pressure applied to the ultrasound probe used to indicate the insertion site in some patients (such as obese and morbidly obese patients) modifies the initial measurement, making this approach inconsistent and unreliable in such cases (16). Additionally, basic ultrasonography may be difficult in people with aberrant spine anatomy (15). Rather than focusing on anticipated epidural space depth, In this work, we concentrated on the direct identification of the epidural space using CFD and we found that the lumbar epidural failure rate might still be as high as 27 - 32% even in the presence of a positive loss of resistance test (17). Additional techniques for determining the epidural space include attaching an epidural balloon to a needle. The following methods have not proven to be better than the conventional loss of resistance technique or the use of color flow Doppler, which is more accurate: Bioimpedance (17), optical reflectometry (18), optical tomography (18), nerve stimulation (19), sound detection (20), syringe plunger pressure detection (21, 22), and non-invasive pressure detection (22). These methods are either costly or unfeasible.

As far as we are aware, this is the first study to use color flow Doppler ultrasound to evaluate the epidural needle's location in hysterectomy surgery. Van den Bosch et al. (23) introduced color flow Doppler ultrasound as a rapid technique for assessing epidural catheter position in laboring patients. Their study, involving 40 patients receiving epidural analgesia, successfully visualized flow in all cases, predominantly at the interspace of insertion. The findings highlight the potential of color flow Doppler ultrasound for swift confirmation of catheter position and troubleshooting epidural analgesia issues.

In a retrospective case series by Elsharkawy et al. (24), the study showed that CFD and M-mode are effective in determining the catheter path (25); in 67.5% and 75% of patients, respectively, successful confirmation of catheter tip location was achieved. In total, 94.5% of patients had suitable dermatomal analgesia. The findings imply that using M-mode ultrasound and CFD may be a practical way to confirm the location of the epidural catheter.

Yoo et al. (7) explored the utility of color flow Doppler ultrasonography in the paramedian sagittal oblique view of the lumbosacral spine for caudal epidural injection (CEI). Their study found that this technique is valid, reliable, and feasible, with a high accuracy level comparable to fluoroscopy-guided CEI. The results suggest that color flow Doppler ultrasonography in the LS-PSOV is easily applicable in clinical settings, providing visual confirmation of CEI solution reaching the desired level in the lumbosacral spine, potentially aligning with our study's focus on enhancing procedural accuracy in gynecological surgeries.

In a pediatric population (i.e., babies), epidural localization using ultrasonography (26, 27) and color flow Doppler (28) has been described. In this population, the non-ossified spine facilitates easy ultrasound penetration. Conversely, our study offers a thorough analysis of the application of color flow Doppler to evaluate epidural catheter placement in adult patients.

Riveros-Perez et al. (11) retrospectively assessed 35 patients undergoing labor combined spinal-epidural (CSE) procedures, confirming epidural needle placement with color flow Doppler (CFD) ultrasonography. Their findings highlight CFD as a valuable method for precisely verifying the location of the epidural needle tip during labor pain management. However, they noted that despite the efficacy of the combined spinal-epidural (CSE) technique, identifying the epidural catheter using CFD can pose challenges.

Moving on to the limitations of the study, several should be acknowledged. Firstly, the study's sample size, while calculated to meet statistical power requirements, may still limit the generalizability of the findings. Additionally, the single-center nature of the study may not fully represent broader demographic and clinical variations. Furthermore, the study primarily focused on procedural aspects, pain outcomes, and safety parameters, without extensively exploring long-term outcomes or patient satisfaction beyond the immediate postoperative period. 

Future research endeavors could address these limitations by conducting multicenter studies with larger and more diverse populations and by incorporating more comprehensive outcome measures. This study's strengths lie in its prospective design, rigorous adherence to ethical standards, and the utilization of CFD as an innovative approach for epidural catheter placement during hysterectomy surgeries. The inclusion of comprehensive outcome measures, including flow visualization, dermatome sensory levels, pain assessments, and safety parameters, contributes to a well-rounded understanding of the implications of CFD in this context. 

The study's meticulous methodology, including the use of ultrasound-guided techniques, standardized procedures, and the employment of a curvilinear ultrasound transducer, adds credibility to the results. Additionally, the study's registration with ClinicalTrials.gov enhances the transparency and accessibility of trial details.

### 5.1. Conclusions

The results suggest that CFD is associated with effective anesthesia coverage, lower reported pain levels, and a high degree of procedural accuracy and safety. While acknowledging its limitations, such as sample size and single-center nature, the study advocates for the integration of CFD into routine clinical practice to enhance procedural outcomes and patient safety. Future research should further explore the long-term implications of CFD-guided epidural anesthesia and consider its applicability in diverse clinical settings.

## Data Availability

Data are available from the corresponding author upon reasonable request.

## Clinical Trial Registration Code:

NCT06019039.
